# Potential Pain in Fish and Decapods: Similar Experimental Approaches and Similar Results

**DOI:** 10.3389/fvets.2021.631151

**Published:** 2021-04-20

**Authors:** Robert W. Elwood

**Affiliations:** School of Biological Sciences, Queen's University, Belfast, United Kingdom

**Keywords:** pain, fish, decapod, nociception, reflex

## Abstract

I review studies that examined the possibility of pain experience in fish and note how they provided guidance on general methods that could be applied to other animals such as decapod crustaceans. The fish studies initially reported the occurrence of prolonged rocking movements in trout and rubbing of their lips if they were injected with acetic acid. Subsequent studies examined the role of morphine in reducing these activities and examined shifts in attention when responding to noxious stimuli. Various studies take up these themes in decapods. The results reported for the two taxonomic groups are remarkably similar and indicate that responses of both go beyond those expected of mere nociceptive reflex. Thus, the idea of pain cannot be dismissed by the argument that fish and decapods respond only by reflex. The responses of both clearly involve central processing, and pain experience, although not proven for either, is a distinct possibility. These studies have been the subjects of highly critical opinion pieces and these are examined and rebutted. The conclusion is that both fish and decapods should be awarded consideration for their welfare.

## Introduction

The seminal paper on rainbow trout (*Oncorhynchus mykiss*) by Sneddon et al. (1) established general methods for investigating pain in animals. It used two main approaches. First, two types of neurons that detected potentially painful stimuli were demonstrated, and their neural responses to specific noxious stimuli were reported. Second, the paper described various behavioral responses to a potentially painful event that could not be simple reflexes. It was this latter approach that provided the greater guidance for work on possible pain in decapod crustaceans. Various receptors had already been found on the antennae of spiny lobsters (*Panulirus argus*), some of which are chemosensory, whereas others responded to mechanical and chemical stimulation (2). Subsequent studies demonstrated receptors in crayfish (*Procambarus clarki*) that respond to high but not low temperatures and appear to function as nociceptors (3). Thus, in my laboratory, we chose not to examine the neurons but, rather, concentrated on asking if the responses to noxious stimuli were reflexive or not. This was a priority because the idea of invertebrates being able to experience pain had long been dismissed because their responses were said to be pure reflexes (4). A reflex is defined here as a short-term reaction to a stimulus without integrating information about other motivational requirements. Responses that are influenced by other sources of information and motivational requirements result from central processing, and swift avoidance learning, and long-term behavioral changes that are likely to enhance future avoidance of tissue damage are not reflexes.

## Abnormal Behavior and Effects of Analgesics and Local Anesthetics

To examine behavioral responses to potentially painful stimuli, Sneddon et al. (1) injected acetic acid, bee venom or saline control into the upper and lower lips, or simply handled rainbow trout in a further control group. Acetic acid and bee venom were selected because they cause pain in humans and are used in pain research in mammals. All groups of fish showed an increase in opercular ventilation rate, but the acetic acid and bee venom produced a greater response, and the elevated rate lasted longer than in the two control groups. This possibly reflects a greater physiological stress response that demands a high oxygen consumption with the potentially painful stimuli. All groups of fish stopped feeding but those of the venom and acetic acid groups avoided ingesting food for longer than did the control groups. That is the noxious stimuli interrupted normal behavior, but the use of a covered shelter in the tank was not affected and neither was the general swimming activity. Another finding was the performance of apparently anomalous activities following noxious treatments. Trout injected with venom or acid performed a rocking movement, where they moved from side to side, balancing on either pectoral fin, while resting on the gravel substrate. Further, the acid group rubbed their lips into the gravel and against the side of the tank. It was this final observation that had the largest impact on my own thoughts on potential pain because it showed a prolonged activity directed at the point at which the noxious stimulus had been applied. Similar directed rubbing is seen in humans and other mammals and it is thought to be a key indicator of pain (5–7). Trout injected with both morphine and acetic acid show much decreased rocking and rubbing compared to those injected with just the acid (8). Injection with a local anesthetic (lidocaine) has a similar effect (9).

This work on rainbow trout formed the basis of my laboratory's first experiment on potential pain in decapod crustaceans (10). We sought to examine the behavioral responses of glass prawns (*Palaemon elegans*) that had acetic acid, sodium hydroxide or seawater brushed onto the distal part of one antenna or that had a crushing force applied *via* forceps. We also sought to determine if a local anesthetic (benzocaine), applied before the noxious stimuli were applied, would modify responses to these treatments. Finally, we asked if any activities directed toward the antennae were preferentially directed at the treated antenna rather than being directed to both. The experiment was conducted in two stages. First, a prawn was removed from a tank and placed on damp tissue paper. One antenna was randomly selected and was brushed with either benzocaine or seawater. The prawn was then placed in a small tank and the behavior observed for 5 min. Second, the prawn was then removed, and the same antenna was subject to one of the three noxious stimuli or seawater control. It was then placed in the observation tank and the behavior again observed for 5 min.

**Figure 1:**
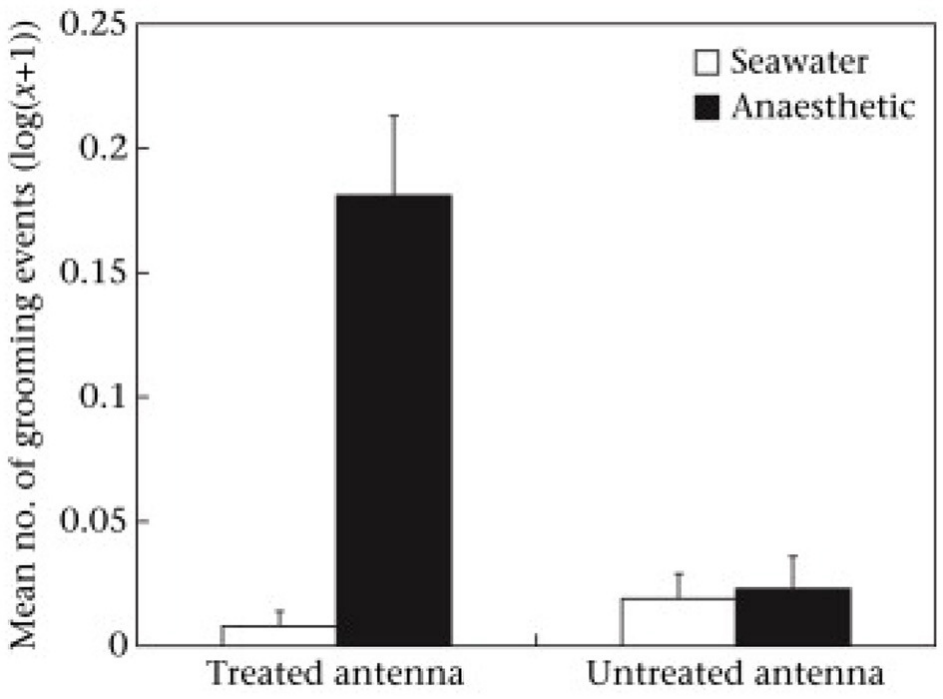
Mean ± SE [log(*x* + 1)] of grooming of treated and untreated antennae following application of seawater or anesthetic in the first observation. Reproduced with permission from Barr et al. (10).

While the benzocaine was applied to the antenna about half the animals showed tail flicking, which is an escape response comprising rapid flexing of the abdomen that would normally propel the animal backwards. Because none of those having seawater applied performed flexing it showed that the benzocaine was initially aversive and presumably stimulated the nociceptors before silencing them in the way expected of a local anesthetic. When the animals were placed into water there was a high level of grooming of the antenna that had been treated with benzocaine, but very little directed to the alternative antenna and very little of either antenna if treated with seawater (Figure 1). Grooming involved repeated pulling of the antenna through the small pincers on the front legs of these animals. That is, the benzocaine did not have an immediate anesthetic effect and appeared to be aversive when first applied. Indeed, local anesthetics are reported to cause pain when first administered to humans, largely due to the acid medium (11), so it seems something similar happens in crustaceans. However, the benzocaine seemed to have the expected anesthetic effect by the time the second treatment was applied because no prawn with benzocaine then showed a tail flick response, but it was seen in those previously treated with seawater. Further, it was only seen in those animals receiving a noxious stimulus (chemical or pinching) and not those whose second treatment was again seawater.

**Figure 2:**
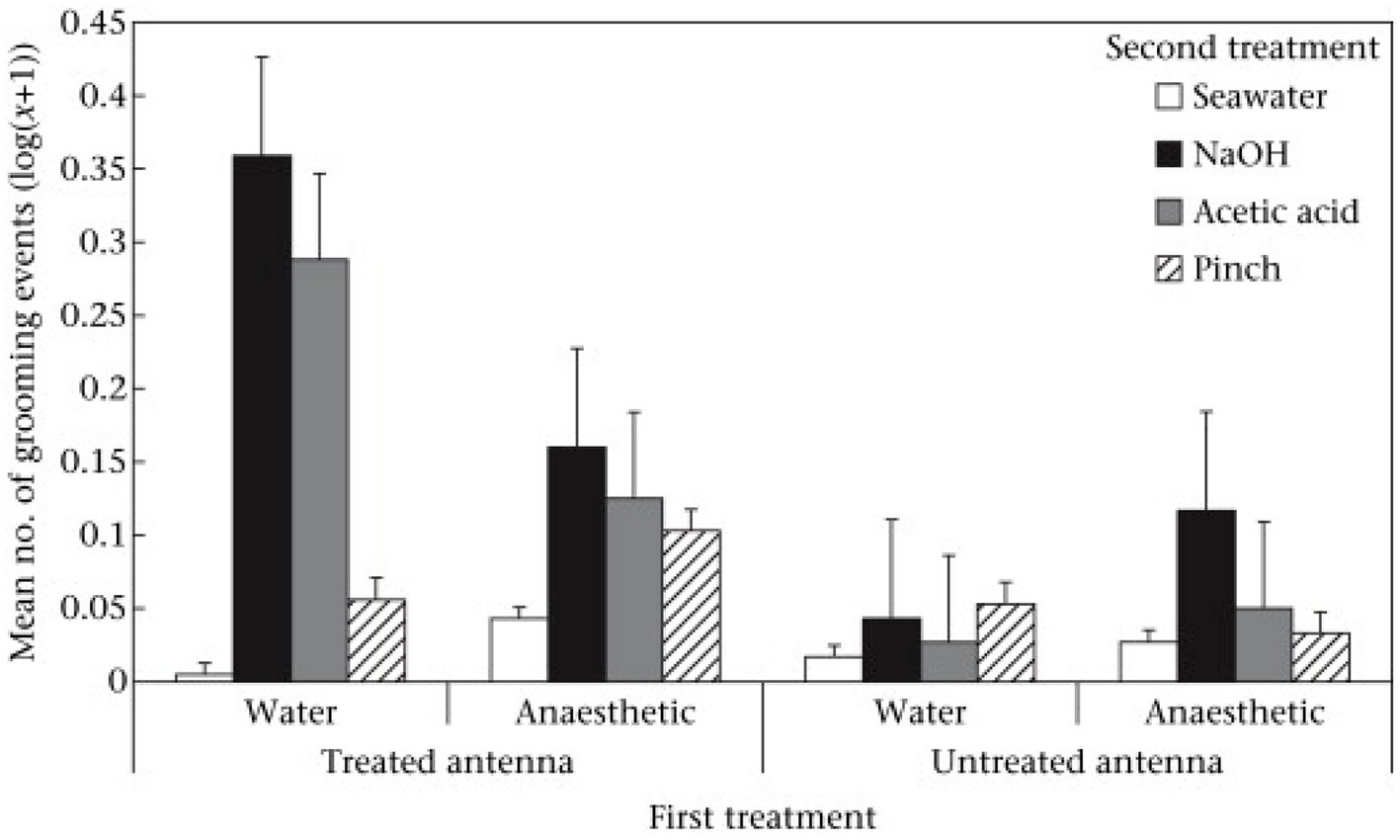
Mean ± SE (log(*x* + 1)) of grooming of treated and untreated antennae in the second observation. Reproduced with permission from Barr et al. (10).

When the prawns were placed again into the tank for the second observation period there were no differences between the groups in their swimming activity. However, there were differences in activities directed at the antennae that were attributable to both the first treatment (benzocaine vs. seawater) and the second treatments (chemicals, pinching, or seawater). In short, grooming was most frequently seen when the second treatment was chemical and applied to an unanesthetized antenna. Further, the grooming was directed specifically at the treated antenna rather than the alternative antenna (Figure 2). Prawns also showed rubbing of the antennae against the side of the tank and again this was of the treated antenna rather than the alternative antenna. It also occurred most with chemical treatment and if the first treatment had been seawater. Thus, we saw two responses involving the antennae that were reduced by pre-treatment with benzocaine and always directed more at the treated antenna compared to the untreated. Pinching with forceps elicited much less of a response than did the chemical treatment but there was more rubbing of the pinched antenna compared to that which was not pinched. However, rubbing was not significantly suppressed by benzocaine.

The results from our experiment on prawns showed remarkable similarities to those on trout (1, 8). Both trout and prawns responded to acetic acid by showing prolonged directed activity toward the site of application and trout had this response reduced by analgesic and prawns by local anesthetic. Further, shore crabs, *Carcinus maenas*, brushed with acetic acid on the mouth parts or an eye showed marked behavioral changes that, in the latter case, involved the specific treated eye (12). The responses of grooming and rubbing seen in these experiments appeared too prolonged and complex to be described as reflexive. Additionally, brown crabs, *Cancer pagurus*, that had a claw removed to simulate the fishery practice of collecting just the claws showed behavior directed at the wound and guarding of the wound when an intact competitor was encountered (13). Thus, there is a similarity with pain responses seen in humans and other mammals and these observations are consistent with the idea of pain. However, one should note that experiments using hydrochloric acid on three species of prawns found no such directed activities (14) and experiments on Atlantic cod, *Gadus morhua*, with acetic acid injection failed to show activities directed at the site of treatment (15).

## Shifts in Attention

Another approach to examine potential pain in fish involved changes in the attention of trout to novel stimuli (16). Trout normally avoid novel objects but did not display this avoidance when they had been injected with acetic acid in the lips. Avoidance was seen, however, in fish injected with saline, and in fish treated with acid plus morphine. It was suggested that the pain caused by the acid dampened the attentiveness of the fish toward a novel item. Thus, it was suggested that a higher order process was involved in the attention decline while the fish was in pain (17). Studies in crayfish (*Procambarus clarki*) that examined responses after the cessation of the noxious stimulus (repeated electric shock) showed enhanced, relatively long-lived, fear responses (18). This finding was described as an enhancement of anxiety and shown to be mediated by elevated serotonin levels. Indeed, injection of serotonin without shock was sufficient to cause anxiety behavior. Remarkably, injection of an anxiolytic, originally produced to reduce anxiety in humans, also reduced anxiety in crayfish. These changes in anxiety and avoidance of risk are particularly interesting because they reduce further tissue damage over the longer term, which is presumed to be the key function of pain (19–21). This protection following noxious stimuli (wounding) has been demonstrated in squid during predation attempts by fish. Squid that were wounded attracted more predation attempts than unwounded squid, and survival was not as high as those without wounds. However, the lowest survival was seen in squid that were given an analgesic as well as the wound (22). It was suggested that the wound increased anxiety and enhanced attention to the predator, but this depended on nociceptive input to the central nervous system. It was consistent with a prediction of the function of pain. Similar findings on improved survival have since been noted in crustaceans (23).

## Long-Term Effects on Motivation and Learning

Long-term changes in behavior after noxious stimuli have also been shown in hermit crabs (*Pagurus bernhardus*) (24, 25). Crabs were given shocks on their abdomen within their gastropod shells whereas control crabs were not shocked. The crabs were subsequently offered a new empty shell and the motivation to obtain a new shell was found to be markedly increased in those exposed to the noxious stimulus. Shocked crabs approached the new shell more quickly, moved to the new shell after a shorter latency, were more likely to change shells, and gave a less thorough investigation of the interior of new shell before moving in. Such shifts in behavior toward shells after shock clearly reflect enhanced motivation to switch shells and suggest that the shell is perceived as being low quality after shock (26, 27). The shift in motivation was found up to 24 h after the noxious stimulus. Thus, the resulting change in behavior could not be called a reflex.

Another way that pain might enhance fitness is that it increases the salience of the noxious stimulus and thus enables rapid avoidance learning. Such learning has been demonstrated in both fish (28) and in shore crabs (*Carcinus maenas*) (29). In the latter example crabs were repeatedly placed in an enclosure containing two dark shelters. In one shelter crabs were shocked if they entered and continued to be shocked if they remained, but the other shelter was safe. Crabs showed a significant shift in their entries to the shelters after just two trials and thus avoided the shock shelter. A further experiment used a different process of exposure to the shock and non-shock shelters (30). Whereas, in the first study the safe and shock shelter were simultaneously present in each trial, in the later study the animal was exposed to one shelter at a time, alternating with safe and shock experiences. Only after five exposures to each were the crabs given a choice of the two, but they showed no preference for the safe shelter. They did, however, reduce the number of shocks received during the trials because they simply left the shock shelter more quickly in later trials. Of course, one might ask why they simply did not refuse to enter either shelter as a means of avoiding shock. The problem with that is that crabs use dark shelters on the shore to avoid predation. Thus, entering a shelter is important for survival but, nevertheless they leave should they receive shock. This is likened to paying a cost to avoid shock. We see this repeatedly in studies in which animals leave a safe shelter such as a shell for hermit crabs (31) or giving up feeding opportunities to avoid the shock in fish (32). Paying a cost to avoid the stimulus is a clear demonstration that the animals find the stimulus aversive, and it is consistent with the idea of pain.

## Trade-Offs With Other Motivational Requirements

Observations of non-reflexive, long-term changes in behavior after noxious stimulation have been influential in our thinking about the occurrence of pain but persuasive experiments have also indicated that some swift responses are not purely reflexive. One way of showing this is to examine if escape from the noxious stimulus is traded-off against other motivational requirements. If they are traded-off, then they must be the result of a higher order decision rather than a reflex (33). There are examples of this with both fish and crustaceans. Trade-off between avoiding an area of electric shock and being near a companion fish has been demonstrated (28) as has that between shock avoidance and the requirement for food (32). Similarly, hermit crabs might leave their gastropod shells if shocked within, however, they evacuate from less preferred species of shell at a lower voltage than a preferred species (31) and, if the voltage is kept constant, they are more likely to get out of the less preferred species (25). Further, hermit crabs are less likely to leave their shell if the odor of a predator is in the surrounding water (34). Fish and crustaceans clearly trade-off various motivational requirements against shock avoidance. These studies demonstrate that even short-term or immediate responses may not be reflex escape responses but rather the result of centrally organized decisions that maximize fitness following tissue damage.

## Physiological Responses

Injury not only triggers behavioral responses that appear to indicate pain, but it also initiates a series of physiological changes that are termed stress responses (35). The main stress response in fish has similarities with those of other vertebrates (36). It is mediated by the hypothalamic-pituitary-interrenal axis and produces a cascade of hormonal changes. A key effect is the metabolism of glycogen to release glucose, thus providing an abundant energy supply to help deal with energetic activities required for fight or flight. An analogous system occurs in crustaceans with the release of crustacean hyperglycemic hormone (37). It too mobilizes intracellular glycogen and converts it to glucose. It also elevates lactate. After injury, such as the fishery practice of twisting and removing a claw, there is a rapid elevation of glucose and lactate in brown crabs (*Cancer pagurus*) (38). A sharp increase in lactate is also seen in shore crabs after a series of electric shocks (39). However, there is a potential problem with the interpretation of this finding because electric shock often induces escape responses, and the high lactate might be due to elevated activity rather than the shock *per se*. To get around this problem Elwood and Adams (39) recorded the behavior of crabs that were shocked and of non-shocked controls. Crabs that showed particularly high activity or particularly low activity were excluded from the data set, so that just those that walked around the enclosure were used in the lactate analysis. There was still a large difference in lactate between the shock and non-shock group, demonstrating that the stress response was specific to the noxious experience of electric shock rather than being mediated by a behavioral change. That is the shock was a stressful stimulus for the crabs.

## Some Taxonomic Differences

The conclusion so far is that behavioral and physiological responses to noxious stimuli are similar in fish and crustaceans. However, they differ in some respects. This is the case with the analgesic effect of morphine in fish (8, 16). With crustaceans, early studies suggested morphine had similar effects. For example, mantis shrimp, *Squilla mantis*, and the crab, *Chasmagnathus granulatus*, showed a reduced response to electric shock if treated with morphine (40, 41). However, *C. granulatus* given morphine also shows a reduced responsiveness to a moving shadow, a stimulus that normally elicits escape or defensive responses (42). Thus, the effect of morphine is not specific to analgesia and could be explained if morphine reduced responsiveness to all stimuli. This was tested by Barr and Elwood (43) using the responses of shore crabs being placed in a brightly lit arena with a single dark shelter. Crabs thus placed typically move into the shelter to escape the light. The experiment had two main factors, some crabs were injected with morphine while others received saline, and some received shock when in the shelter while others did not. Each crab had 20 trials with the shelter in the experiment. The rationale is that crabs might be expected to avoid the shelter if they receive shocks or at least hesitate before entry, however, those pre-treated with morphine should show less aversion of the shelter because the morphine should have an analgesic effect. The results did not uphold this prediction. During the early trials few of the crabs that had been given morphine entered the shelter irrespective of receiving shocks or not. They appeared to be limp, unable to move and unresponsive. However, over the course of the first 10 trials, the crabs with morphine recovered their responsiveness and started to move into the shelter but were not more likely to enter and did not enter more quickly than crabs without morphine. Thus, no analgesic effect was found, rather morphine causes the animals to become unresponsive to all stimuli and this lasts for a short period, a finding that would account for the apparent effect in *S. mantis* and *C. granulatus*.

A second difference in responses between crustaceans and fish occurs with capsaicin. Capsaicin causes a burning sensation in humans and appears to cause pain in most mammals, but not birds (44). When capsaicin or acetic acid were injected into the lips of cod there were similar behavioral changes, indicating that fish too have capsaicin receptors (15). By contrast, neither crayfish, *P. clarkii* (3) nor shore crabs, *C. maenas* (12) showed any responses to application of capsaicin. This variation in receptivity is dependent upon relatively minor differences in the molecular structure of vanilloid receptors that typically respond to high temperature, and some chemicals, but the ecological reasons for this variation is not clear (45). However, these minor differences have little or no effect on the evidence of behavioral and stress responses to noxious stimuli that are consistent with pain in fish and crustaceans.

## Opposition to the Idea of Pain in Fish and Decapods

The experimental studies noted above have elicited fierce resistance from some authors (46–49). It is claimed that the experimental work has been “mission oriented” and has not used the “detached tradition expected of basic science” (46). Rose et al. (46) further claimed that it is often “faith-based research” and that “these biases have an insidious impact on the credibility of the “science” surrounding aquatic animal welfare.” It should be noted, however, that the early work on fish pain was funded by the UK Biotechnology and Biological Sciences Research Council, whose teams of expert reviewers only support science of the highest rigor. The application process for that project was described in Braithwaite (17).

Diggles (49) states “scientific claims that fish or crustaceans “may feel pain” have been largely based on a few dubious and disputed studies done on a small number of animals and species.” For my own part, I have published 13 experiments that relate to decapod welfare, using four species, in 10 papers. The mean sample size per experiment was 91.7 (range 40–244); the chosen numbers were dependent on the complexity of the experimental designs and the requirements of the proposed statistical analyses. These are not small numbers of animals. Other authors have used different decapod species so the additional claim of a few species by Diggles is unfounded.

A key aim of these highly critical opinion pieces is to prevent changes to fishery operations (46–48). However, these attempts to reject welfare improvements have, in turn, been heavily criticized [e.g., (33, 50–52)]. Rather than go through the detailed arguments made by each side I refer here to a few major objections.

It has been suggested that because we know the brain areas in humans that have been linked to the experience of pain then any animal lacking these areas cannot experience pain (46, 47). This contention is based on “the bioengineering principle that structure determines function” (47). Because fish and decapods do not have these human structures the idea of pain is dismissed. There is a complete denial that very different brains can have similar functions as has been noted for visual ability in humans, cephalopods, and decapods (21). We also see olfactory abilities in very different animals despite having very different brain morphology. Further, it has been stated that brain size is related to sentience and that those of fish and crustaceans are too small for the necessary neural computation. It should be noted, however, that the very surprising, complex cognitive abilities of bees is achieved with a very small brain (53). Such studies in comparative cognition have led to the idea that a “bottom-up perspective” might lead to a better understanding of basic building blocks of specific advanced functions (54). It is that approach reviewed here that should help our understanding of other mental capacities such as the ability to feel pain. Brains comprise many neurons, each with many dendrites, which enable a vast number of connections, distinct circuits, and functional compartmentalisations. It is those connections, rather than the embryological derivation of brain regions, that are important for computational complexity and function. Viewed in this way the similarity of function and abilities across taxa appears less surprising, and the possibility of pain in fish and decapods cannot be dismissed (21, 50, 51).

There is a repeated claim that the responses of fish and decapods to noxious stimuli are merely the result of nociceptive reflexes that do not require higher order processing (46). However, the experimental studies noted above unequivocally demonstrate that the responses to noxious stimuli cannot be explained by reflex alone. Rather, the animals respond by integrating information from different sources, and that requires central processing. They also show prolonged shifts in responsiveness that provide long-term protection from further damage, again that cannot be a reflex (33).

Key (47) states that human pain involves conscious neural processing and there are repeated calls for conscious feeling to be demonstrated when suggesting pain in animals (46–48). However, consciousness is known as the “hard problem” because it is impossible to demonstrate (55), and these demands for the impossible to be shown has caused confusion. For example, Stevens et al. (48) demanded that “conscious higher level neural processing” must be demonstrated to support the idea of pain. A few lines later, however, the authors retreat from that demand and state “We agree with Dawkins that one should address animal welfare pragmatically using stress-related indicators without reference to conscious experiences.” This retreat is welcome because the numerous experimental studies noted in the present article have consistently used pragmatic indicators without reference to consciousness.

## Conclusions

Responses to noxious stimuli that are consistent with the idea of pain are seen in both fish and crustaceans (7). It is important to note, however, that there is no conclusive proof of pain in any animal (33). Conversely, Key (47) acknowledges that he cannot prove that fish do not feel pain. Thus, pain in these taxa can neither be proven nor disproven but, in that case, we must at least accept the possibility of pain (21, 56). Given the large number of studies that are consistent with the idea of pain, that possibility is much higher than if those studies had not been consistent. If pain is possible, then the precautionary principle should be invoked (57). That is, although we accept that there is no absolute proof, we take measures to ensure that animals do not suffer by our actions, just in case. That approach is not questioned with respect to mammals and is increasingly accepted for fish and now for decapods. For example, there has been a recent legal change in Switzerland banning some slaughter methods for lobsters and crabs, and the British Veterinary Association now accept that decapods are sentient and calls for stunning before killing (58, 59). These moves suggest that the experimental work on both decapods and fish are not widely regarded as “dubious” or “faith-based,” and that both taxa will soon gain further protection.

## Author Contributions

The author confirms being the sole contributor of this work and has approved it for publication.

## Conflict of Interest

The author declares that the research was conducted in the absence of any commercial or financial relationships that could be construed as a potential conflict of interest.
